# The effects of microRNAs on human neural stem cell differentiation in two- and three-dimensional cultures

**DOI:** 10.1186/scrt437

**Published:** 2014-04-11

**Authors:** Lara Stevanato, John D Sinden

**Affiliations:** 1 ReNeuron Ltd., Surrey Research Park, 10 Nugent Road, Guildford, Surrey GU2 7AF, UK

## Abstract

**Introduction:**

Stem cells have the ability to self-renew or to differentiate into numerous cell types; however, our understanding of how to control and exploit this potential is currently limited. An emerging hypothesis is that microRNAs (miRNAs) play a central role in controlling stem cell-fate determination. Herein, we have characterized the effects of miRNAs in differentiated human neural stem cells (hNSCs) by using a cell line currently being tested in clinical trials for stroke disability (NCT01151124, Clinicaltrials.gov).

**Methods:**

HNSCs were differentiated on 2- (2D) and 3-dimensional (3D) cultures for 1 and 3 weeks. Quantification of hNSC differentiation was measured with real-time PCR and axon outgrowth. The miRNA PCR arrays were implemented to investigate differential expression profiles in differentiated hNSCs. Evaluation of miRNA effects on hNSCs was performed by using transfection of miRNA mimics, real-time PCR, Western blot, and immunocytochemistry.

**Results:**

The 3D substrate promoted enhanced hNSC differentiation coupled with a loss of cell proliferation. Differentiated hNSCs exhibited a similar miRNA profiling. However, in 3D samples, the degree and timing of regulation were significantly different in miRNA members of cluster mi-R17 and miR-96-182, and hsa-miR-302a. Overall, hNSC 3D cultures demonstrated differential regulation of miRNAs involved in hNSC stemness, cell proliferation, and differentiation.

The miRNA mimic analysis of hsa-miR-146b-5p and hsa-miR-99a confirmed induction of lineage-committed progenitors. Downregulated miRNAs were more abundant; those most significantly downregulated were selected, and their putative target mRNAs analyzed with the aim of unraveling their functionality. In differentiated hNSCs, downregulated hsa-miR-96 correlated with SOX5 upregulation of gene and protein expression; similar results were obtained for hsa-miR-302a, hsa-miR-182, hsa-miR-7, hsa-miR-20a/b, and hsa-miR-17 and their target NR4A3. Moreover, SOX5 was identified as a direct target gene of hsa-miR-96, and NR43A, a direct target of hsa-miR-7 and hsa-mir-17 by luciferase reporter assays. Therefore, the regulatory role of these miRNAs may occur through targeting NR4A3 and SOX5, both reported as modulators of cell-cycle progression and axon length.

**Conclusions:**

The results provide new insight into the identification of specific miRNAs implicated in hNSC differentiation. These strategies may be exploited to optimize *in vitro* hNSC differentiation potential for use in preclinical studies and future clinical applications.

## Introduction

Stem cell research has the potential to support future medical advances, especially in the unmet need for chronic diseases [1]. Stem cells play central roles, both in development of the organism and repair of damaged tissue. CTX0E03 is a clonal conditionally immortalized human neural stem cell (hNSC) line [2]. This cell line has defined quality characteristics that are required for cell banking under good manufacturing practice (GMP), to ensure reliable and reproducible stocks of cells suitable for clinical application [3]. HNSCs can differentiate into neurons, glia, and oligodendrocytes and have been shown to ameliorate neurologic deficits in a rodent model of focal ischemia after transplantation into the brain [2,4,5]. Recently this hNSC line was tested in a human clinical trial for stroke disability in Scotland, the PISCES trial (NCT01151124, Clinicaltrials.gov). Although the functional properties of hNSCs have been studied extensively, the molecular mechanisms underlying neural stem differentiation are not fully understood.

MiRNAs have received emerging attention over the last years as significant regulatory molecules [6]. They constitute a subpopulation of small RNAs of average 22 nucleotides in length. Unlike messenger RNAs (mRNAs), miRNAs do not encode proteins, but rather bind 3′ untranslated region (3′ UTR) of mRNAs, regulating their stability and translation into proteins. Functional studies indicate that miRNAs participate in the regulation of a number of cellular processes, including development, proliferation, and differentiation [7]. The discovery of miRNAs has offered new potential for modulating stem cell lineage commitment and differentiation by posttranscriptional gene regulation [8,9]. Many studies have demonstrated that transient overexpression or inhibition of brain-specific miRNAs in stem cells significantly directed their differentiation toward neuronal cell lineages [10]. Several miRNAs have been implicated in regulating self-renewal of neural stem cells and neuronal fate specification [11]. Herein we compared miRNA profiling obtained from *in vitro* assays of a clinical grade hNSC line to investigate further miRNA functionality and effects on neuronal and glial differentiation potential.

In two-dimensional (2D) standard differentiation protocols, the complexity of the *in vivo* environment is not reflected, and consequently, the induction and regulation of hNSC differentiation is not optimal. *In vivo*, cells are surrounded by other cells as well as many extracellular ligands, including many types of collagens, laminin, and other matrix proteins (extracellular matrix, ECM) in a three-dimensional (3D) microenvironment. Previous studies showed that specific topologic architecture of the materials mimicking ECMs and their geometry influence cell phenotype and fate [12-19]. A number of commercially available 3D culture systems are available, including Alvetex (Amsbio, UK). Alvetex is a highly porous polystyrene scaffold engineered with a well-defined and uniform architecture into a 200-μm-thick membrane, which provides a 3D space into which cells can invade and differentiate [20]. The scaffold is formed by polymerization in a biphasic emulsion, consisting of an aqueous and a nonaqueous monomer/surfactant phase, termed a high-internal-phase emulsion, or HIPE [21,22]. The resulting polymer (poly-HIPE) consists of a relatively homogeneous porous network of voids, linked by interconnecting pores. Moreover, such scaffolds have been proven amenable for 3D *in vitro* cell culture [20].

In the present study, 2D and 3D differentiation assays were used to monitor mRNAs of neuronal and glial markers and neurite outgrowth to identify miRNAs involved in the regulation of neuronal/glial differentiation processes.

## Material and methods

### HNSC derivation, culture, and differentiation

CTX0E03 is a fully manufactured conditionally immortalized hNSC line, originally derived from ethically sourced human fetal brain cortical tissue of 12 weeks’ gestation and described in [2]. To set up differentiation assays, a single-cell suspension of hNSCs, obtained from passage 30 to 36 cell cultures, was achieved by trypsinization, and the number of cells determined by using a hemocytometer. Cells were seeded either on standard cell-treated plastic vessels (BD Biosciences) or on polystyrene scaffolds (Alvetex®, Amsbio) in serum-free medium. Cells were maintained at 37°C in a humidified, 5% CO_2_ incubator for 1 and 3 weeks, and cultured in the same defined medium without the mitogens, EGF, bFGF, and 4-OHT (Sigma).

### Measurement of axon-process outgrowth

Measurement of axon-process outgrowth was performed on differentiated and undifferentiated hNSCs fixed with 4% paraformaldehyde (PFA; Pioneer Research Chemicals) and incubated with β3-tubulin (TUBB3) primary antibody (Sigma). After rinsing, the cells were incubated with anti-mouse Alexa Fluor 568 (Invitrogen) secondary antibody. Cell nuclei were counterstained with Hoechst (Sigma, 1 μ*M*). Measurement of axon-process length was carried out on a minimum of three representative fields by using Image-Pro Plus 7 software (Media Cybernetics).

### Real-time RT-PCR (qRT-PCR) analysis of mRNA

HNSC total RNA was isolated by using miRNeasy (Qiagen), according to the manufacturer’s protocol. A minimum of 250 ng of total RNA was reverse-transcribed into first-strand cDNA by using a mix of random primer and poly-dT. Reverse transcription was performed with superscript II reverse transcriptase (Invitrogen) for 1 hour at 42°C, inactivated for 15 minutes at 70°C, and cooled to 4°C. Two microliters of cDNA were used in a PCR reaction containing 2× Roche master mix, 0.1 μg of human universal probe library (UPL; Roche), and 0.4 μ*M* primers for neuronal markers: β3-tubulin (TUBB3), a well-established neuron-specific marker expressed by neuronal precursors [23]; doublecortin (DCX), a marker expressed in developing neurons, and increasingly used as a marker for neurogenesis [24]; microtubule-associated protein 2 (MAP2), involved in microtubule assembly, which is an essential step in neurogenesis [25], and glial markers: glial fibrillary acidic protein (GFAP), a well-recognized glia marker; S100 calcium-binding protein B (S100B), a glia-specific marker expressed primarily by astrocytes; and galactocerebroside (GALC), expressed by differentiating oligodendrocyte precursor cells [26]. The following primer sequences and UPL were used for each markers: TUBB3 (NM_006086), F) gcaactacgtgggcgact, R) cgaggcacgtacttgtgaga, UPL 78; DCX (NM_000555.2) F) gtggaggctggtaaagagca R) aggcccaagcataaggaaat, UPL 6; MAP2 (NM_031845.2), F) cgaactttatattttaccacttccttg, R) ccgttcatctgccattcttc, UPL 2; GFAP (NM_002055.3) F) ccagttgcagtccttgacct, R) tctccagggactcgttcgt, UPL 88; S100B (NM_006272.2) F) caggatccttgcctccaac R) ctcagagcccccggtagt UPL 67; GALC (NM_001037525.1), F) tggtgcctctttgcatatttta, R) atgtgggagggctcagtg, UPL 9. QRT-PCR results were expressed as relative quantification based on the 2^-∆∆ct^ method [27] and normalized against average of ATP5B and YWHAZ (PrimerDesign) housekeeping genes. A minimum of three biologic replicants was evaluated for each marker and condition.

### Measurement of cell proliferation

Measurement of cell proliferation was performed on differentiated and undifferentiated hNSCs fixed with 4% PFA and stained with Ki67 primary antibody, 1:100, (Thermo Scientific). After rinsing, the cells were incubated at room temperature with anti-rabbit Alexa Fluor 568 (Invitrogen) secondary antibody. Cell nuclei were counterstained with Hoechst (Sigma, 1 μ*M*). A minimum of three representative fields was analyzed for each condition.

### Stem cells and developmental pathways-focused miRNA PCR-array analysis

The stem cells and developmental pathways-focused miRNA PCR array (Qiagen, Sabiosciences) was carried out according to manufacturer’s instructions. For each array, a minimum of 250 ng total RNA was retrotranscribed by using miScript II RT Kit (Qiagen) according to the manufacturer’s instruction. Each condition was run in triplicate. Array analysis was performed by using miScript miRNA PCR-array data analysis [28].

### MiRNA mimic transfection

Before transfection, hNSCs were seeded into a 24-well plate. MiRNA-transfection optimization was performed by using allstars negative control siRNA AF 488 (Qiagen); by using the following condition, the miRNA transfection efficiency was found to be 100%. Each of three miRNA mimics (5 n*M*; Qiagen), hsa-miR-146b-5p, UGAGAACUGAAUUCCAUAGGCU, hsa-miR-23b, AUCACAUUGCCAGGGAUUACC, and hsa-miR-99a, AACCCGUAGAUCCGAUCUUGUG was combined with HiPerFect (Qiagen) according to manufacturer’s instructions. Control samples were transfected with green fluorescent protein (GFP) plasmid. Samples were collected after 1 week. MiRNA-mimic cell internalization after transfection was measured by qRT-PCR using miScript PCR Starter kit (Qiagen) according to manufacturer’s instructions. A minimum of three biologic replicants was evaluated for each marker and condition.

### Computational target gene predictions, validation by real-time RT-PCR, and pathway analysis

DIANA-microT 3.0 algorithm [29,30] was used to identify miRNA target prediction mRNA. In brief, the DIANA-microT 3.0 algorithm consists of (a) alignment of the miRNA driver sequence on the 3′ UTR of a protein-coding gene, (b) identification of putative miRNA recognition elements score (MREs), based on specific binding rules, (c) scoring of individual MREs according to their binding type and conservation profile, (d) calculation of an overall miRNA target gene (miTG) score through the weighted sum of all MRE scores lying on the 3′ UTR. The program is designed to use up to 27 different species to estimate MRE conservation scores and combines both conserved and nonconserved MREs in a final miTG score. Similar analysis was conducted by using PicTar [31,32] and TargetScan [33,34]. QRT-PCR was performed as described by using the following primers: SRY (sex-determining region Y)-box 5 (SOX5, NM_006940.4), F) tttacctcaggagtttgaaagga, R) gcttgtcaccatggctacct, UPL 38; forkhead box N3 (FOXN3, NM_005197.3) F) cattaagaggtgtggcgttttt, R) gacacatgaaccgccactt, UPL 3; nuclear receptor subfamily 4, group A, member 3 (NR4A3, NM_173199) F) tctcagtgttggaatggtaaaaga, R) ggtttggaaggcagacgac, UPL 52; dual-specificity phosphatase 10 (DUSP10, NM_007207.4) F) tgaatgtgcgagtccatagc R) tggcaattcaagaagaactcaa UPL 22; translation initiation factor 4 gamma, 3 (EIF4G3, NM_003760.4) F) attctcaaaacttaaattcaagaagga, R) tttcttccatgtctttggtacagt, UPL 33.

MiRNA KEGG pathway analysis of selected miRNAs was performed by using DIANA LAB [30,35].

### Western blot analysis

HNSC cell monolayers were lysed with 1× SDS sample buffer and dithiothreitol (DTT) reducing agent (AMS Biotechnology), and loaded onto a PAGEgel (Invitrogen). After electrophoresis, the proteins were blotted on nitrocellulose membrane. Immunodetection was performed by using rabbit anti-SOX5 and anti-Nor (NR4A3) polyclonal antibodies (Santa Cruz Biotechnology Inc.) and detected by using a horseradish peroxidase–conjugated anti-rabbit antibody (Cell Signaling Technology). The nitrocellulose membrane was then processed by using chemiluminescence-detection reagents (Thermo Scientific). The blots were stripped and reprobed by using anti-α-tubulin (Sigma, 1:1,000) to act as an internal loading-level standard. Western blot images were captured by using BioRad FluorS Imaging.

### Reporter plasmid transfection and dual luciferase assay

Transient transfections of HeLa cells were performed by using Lipofectamine 2000 (Invitrogen) according to the manufacturer’s instructions. For the luciferase assay, cells were plated at a density of 10^5^ cells/well in 24-well plates and co-transfected with either 100 ng of MiTarget MicroRNA 3′ UTR Target Clone HmiT019538-MT01 (GeneCopoeia, NR4A3 3′ UTR) or HmiT017632-MT01 (GeneCopoeia, SOX5 3′ UTR), as well as 20 n*M* miRNA mimics (hsa-miR-96-5p for SOX5 3′ UTR, and hsa-miR-7-5p, and hsa-miR-17-5p for NR4A3 3′ UTR, respectively) per well. Control wells were transfected with either HmiT019538-MT01 or HmiT017632-MT01 plasmid, and allstars negative-control siRNA AF 488 (Qiagen). Transfection efficiency was found to be 100%. HmiT019538-MT0 and HmiT017632-MT01 plasmids express both firefly and renilla luciferase. Firefly and renilla luciferase activities were measured 24 hours after transfection by using the Luc-Pair miR luciferase assay (GeneCopoeia) and a GloMax 96 Microplate Luminometer (Promega). Firefly luciferase activity was normalized to renilla luciferase activity for each transfected well. In all the experiments, transfection and luciferase assays were performed in triplicate.

### Statistics

Data were analyzed by Student *t* test, and *P* < 0.05 was considered statistically significant. All error bars indicate ± standard deviation of the mean (SDM). In stem cells and developmental pathways focused miRNA PCR array analysis, significant changes were defined as ±1.5-fold up- and down-regulation to a statistically significant extent. A two-sample Student *t* test was used to analyze each comparison.

## Results

### Quantification of differentiation by axon outgrowth and real-time PCR molecular analysis, and quantification of cell proliferation

**Figure 1:**
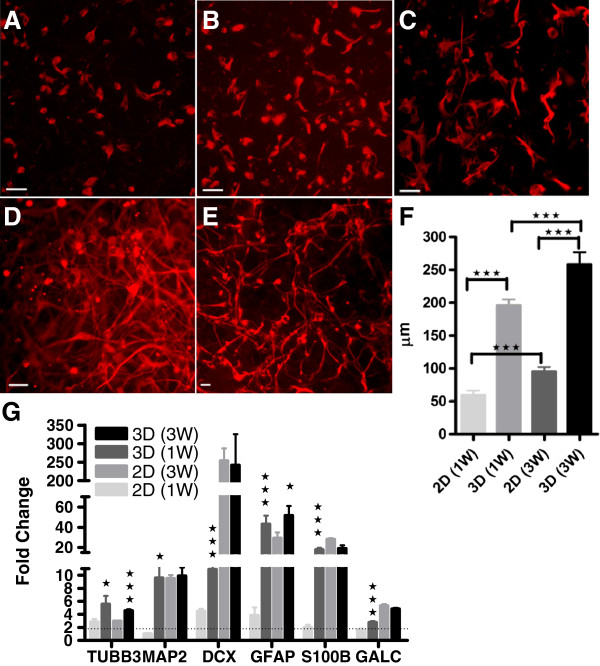
**Quantification of hNSC differentiation. (A-E)** Representative image of **(A)** undifferentiated, **(B)** 1 week (1W) 2D differentiated, **(C)** 3-week (3W) 2D differentiated, **(D)** 1W 3D differentiated, and **(E)** 3W 3D differentiated hNSCs; scale bar, 50 μm. **(F)** Quantification of axon length on 1W and 3W differentiated hNSCs cultured on 2D and 3D substrates. **(G)** QRT-PCR molecular analysis for neuronal (TUBB3, DCX, and MAP2) and glial (GALC, GFAP, and S100B) markers performed on hNSCs differentiated on 2D and 3D for 1W, and 3W, and expressed as fold change compared with undifferentiated control. Statistical analysis showed significant differences between 2D versus 3D samples at the same time point; ± SDMs. *P < 0.05; **P < 0.001, ***P < 0.005, Student t test.

Assessment of the ability of hNSCs to differentiate into neurons, glia, and oligodendrocytes by immunocytochemistry has previously been reported [2]. In the present study, quantification of hNSC differentiation was performed by measuring axon outgrowth and neural/glial marker transcript expression. Axon outgrowth (Figure 1A through E) was significantly enhanced in 3D cultures (195.76 μm ± 9.36, and 258.22 μm ± 18.55) compared with traditional flat-surface cultures (2D) (59.33 μm ± 7.00, and 95.12 μm ± 7.15) evaluated at 1- (1W) and 3- (3W) week differentiation, respectively (Figure 1F). GALC, GFAP, TUBB3, S100B, DCX, and MAP2 mRNA expression-level analysis showed a significant increase in all the tested markers in 3D 1W compared with 2D 1W differentiated cultures. Furthermore, TUBB3 and GFAP were significantly enhanced in 3D compared with 2D differentiated cultures at the 3-week time point (Figure 1G).

**Figure 2:**
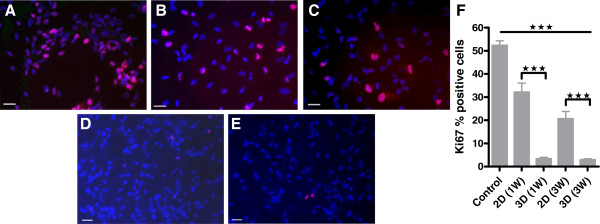
**Evaluation of hNSC proliferation. (A-E)** Representative image of **(A)** undifferentiated, **(B)** 1W 2D differentiated, **(C)** 3W 2D differentiated, **(D)** 1W 3D differentiated, and **(E)** 3W 3D differentiated hNSCs stained with Ki67, marker of cell proliferation; scale bar, 50 μm; Ki67^+^ hNSCs (red), nuclear Hoechst counterstain (blue). **(F)** Quantification of cell proliferation measured as percentage of cells positive for Ki67. Statistical analysis showed a significant reduction in proliferation in 2D and 3D cultures compared with the proliferative control and between 2D and 3D samples at the same time point; ±SDMs. *P < 0.05, **P < 0.001, ***P < 0.005, Student *t* test.

The expression of Ki67, a cellular marker for proliferation [36] (Figure 2A through E), was measured in proliferative hNSC (control) and after differentiation for 1W and 3W on both 2D and 3D cultures. A significant decrease in Ki67 staining was observed in all differentiated samples. The percentage of Ki67 positive cells significantly dropped from 52.28 ± 2.03 in control samples to 32.09 ± 3.96, (1W), 20.59 ± 0.50 (3W) in 2D, and 3.52 ± 0.49 (1W) and 2.86 ± 0.50 (3W) in 3D differentiated cultures (Figure 2F).

### Human cell differentiation and development miScript miRNA PCR array profiles

Stem cells and developmental pathways focused miRNA PCR array (Sabioscience; Qiagen) was implemented to investigate miRNAs differential expression profiles after hNSC 2D and 3D differentiation for 1W and 3W. The geo-mean of SNORD61, SNORD68, SNORD72, SNORD95, SNORD96A, and RNU6-2 was used for data analysis based on the 2^-ΔΔct^ methods. Significant changes were defined as ±1.5-fold up- and downregulation compared with control, undifferentiated/proliferative cultures.

**Figure 3:**
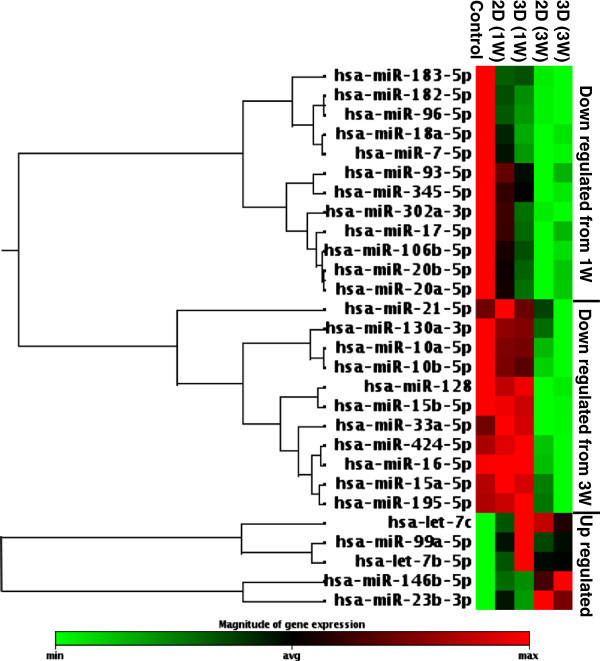
**Human cell differentiation and development miScript miRNA PCR array profiles.** Group clustergram analysis of miRNA PCR array profiles analysis of differentially regulated miRNAs identified in control (undifferentiated hNSCs) and hNSCs seeded on 2D and 3D substrates and differentiated for 1W and 3W.

A total of 84 miRNAs was analyzed in the miRNA PCR array. Hsa-miR-146b-5p, hsa-miR-99a-5p, and the hsa-let-7 family (hsa-let-7c, and 7b), and hsa-miR-23b were identified as significantly upregulated in both 2D and 3D differentiated samples (Figure 3, Table 1). The let-7 family [37] and hsa-miR-23b [38] are known posttranscriptionally to regulate neural cell specification. Interestingly, hsa-miR-99a and hsa-miR-146b-5p, reported to be related to the immune system and cancer inhibition [39,40], have not yet been correlated with neuronal or glial differentiation. The miRNA profiling identified several significantly down-regulated miRNAs: to facilitate data interpretation they were grouped by functionality (Table 2), time, and type of culture substrate. MiRNAs associated with maintenance and regulation of pluripotency, neuronal lineage specification and differentiation [41] and those acting on proteins associated with cell cycle regulation, proliferation, and stem cell renewal [42-46] were identified (Table 2). Time dependency downregulation was observed. Hsa-miR-302a, cluster miR-17-92, and miR-96-182, from 1W, and miR-15 family from 3W were respectively significantly downregulated (Figure 3, Table 1). Significant differences comparing 2D and 3D substrate types were observed at both time points in miRNAs belonging to cluster miR-17-92 [46] and miR-96-182 [47] (regulators of cell proliferation), and hsa-miR-302a (maintenance of stemness), (Figure 3, Table 1).

**Table 1:** Statistical analysis performed on miRNA PCR arrays

	***P*-values (compared with control group)**				***P *** **values (compared with 2D versus 3D)**		
**Mature ID**	**2D (1W)**	**3D (1W)**	**2D (3W)**	**3D (3W)**	**(1W)**	**(3W)**	**Regulation**
hsa-miR-302a-3p	✶	✶✶✶	✶✶✶	✶✶✶	✶✶✶	✶✶	**Down**
hsa-miR-10a-5p	**NS**	**NS**	✶✶✶	✶✶✶	✶✶✶	✶	
hsa-miR-10b-5p	**NS**	✶	✶✶✶	✶✶✶	**NS**	**NS**	
hsa-miR-96-5p	✶✶✶	✶✶✶	✶✶✶	✶✶✶	✶✶✶	✶✶	
hsa-miR-183-5p	✶✶✶	✶✶✶✶	✶✶✶	✶✶✶	✶✶✶	✶✶✶	
hsa-miR-182-5p	✶✶✶	✶✶✶	✶✶✶	✶✶✶	✶	**NS**	
hsa-miR-7-5p	✶✶✶	✶✶✶	✶✶✶	✶✶✶	✶✶	**NS**	
hsa-miR-17-5p	✶✶✶	✶✶✶	✶✶✶	✶✶✶	✶✶✶	✶✶✶	
hsa-miR-18a-5p	✶✶✶	✶✶✶	✶✶✶	✶✶✶	✶✶✶	✶✶✶	
hsa-miR-20a-5p	✶✶	✶✶✶	✶✶✶	✶✶✶	✶✶✶	✶✶✶	
hsa-miR-20b-5p	✶✶✶	✶✶✶	✶✶✶	✶✶✶	✶	✶✶✶	
hsa-miR-93-5p	✶✶✶	✶✶✶	✶✶✶	✶✶✶	✶✶✶	✶✶	
hsa-miR-106b-5p	✶✶✶	✶✶✶	✶✶✶	✶✶✶	✶✶✶	✶✶	
hsa-miR-15a-5p	**NS**	**NS**	✶✶✶	✶	**NS**	**NS**	
hsa-miR-15b-5p	**NS**	**NS**	✶✶✶	✶✶✶	**NS**	**NS**	
hsa-miR-16-5p	**NS**	**NS**	✶✶✶	✶✶	**NS**	**NS**	
hsa-miR-195-5p	NS	✶	✶✶✶	✶✶	**NS**	**NS**	
hsa-miR-21-5p	✶	**NS**	**NS**	✶✶	✶✶✶	✶✶	
hsa-miR-33a-5p	**NS**	**NS**	✶✶✶	✶✶	**NS**	**NS**	
hsa-miR-128	**NS**	**NS**	✶	✶	**NS**	**NS**	
hsa-miR-424-5p	**NS**	**NS**	✶✶	✶✶✶	✶✶✶	**NS**	
hsa-miR-130a-3p	**NS**	✶✶	✶✶✶	✶	**NS**	**NS**	
hsa-miR-345	✶	✶✶✶	✶✶✶	✶✶✶	**NS**	**NS**	
hsa-let-7b	✶✶✶	✶✶✶	✶✶✶	✶✶✶	✶✶✶	**NS**	**Up**
hsa-let-7c	✶✶✶	✶✶✶	✶✶✶	✶✶✶	✶✶✶	✶✶✶	
hsa-miR-146b-5p	✶✶✶	✶	✶✶✶	✶✶✶	✶✶✶	**NS**	
hsa-miR-23b	✶✶✶	✶✶✶	✶✶✶	✶✶✶	✶✶✶	**NS**	
hsa-miR-99a	✶✶✶	✶✶✶	✶✶✶	✶	✶✶✶	**NS**	

Data were analyzed with Student *t* test, ✶ *P* < 0.05, ✶✶*P* < 0.001, ✶✶✶ *P* < 0.005, and nonsignificant (NS).

**Table 2:** Functions of downregulated miRNAs

**Mature ID**	**Family/Cluster**	**Function**	**Reference**
hsa-miR-302a-3p		**Maintenance and regulation of pluripotency**	[48,49]
hsa-miR-10a-5p			
hsa-miR-10b-5p			
hsa-miR-424-5p			
hsa-miR-130a-3p			
hsa-miR-345			
hsa-miR-96-5p	**miR-96-182**	**Neuronal lineage-specification and differentiation**	[41,47]
hsa-miR-183-5p			
hsa-miR-182-5p			
hsa-miR-7-5p			
hsa-miR-128			
hsa-miR-17-5p	**miR-17/miR17-92**	**Acting on proteins associated with cell-cycle regulation, proliferation, and stem cell renewal**	[42-46]
hsa-miR-18a-5p			
hsa-miR-20a-5p			
hsa-miR-20b-5p			
hsa-miR-93-5p			
hsa-miR-106b-5p			
hsa-miR-15a-5p	**miR-15**		
hsa-miR-15b-5p			
hsa-miR-16-5p			
hsa-miR-195-5p			
hsa-miR-21-5p			
hsa-miR-33a-5p			

### Mimic miRNA transfection and real-time RT-PCR quantification of neuronal and glial gene markers

**Figure 4:**
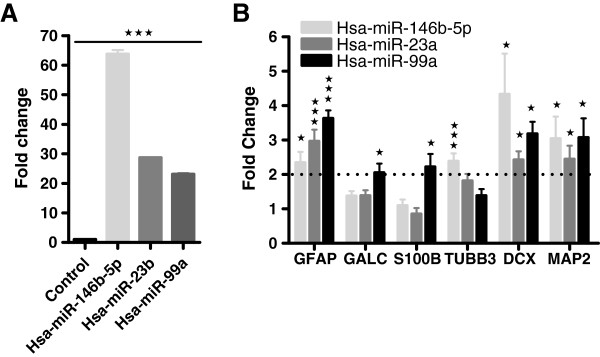
**MiRNA mimic transfection analysis. A)** hsa-miR-146b-5p, hsa-miR-23b, hsa-miR-99a transfected mimics were measured by real-time RT-PCR, and expressed as fold change compared with GFP transfected hNSCs (control). **B)** QRT-PCR molecular analysis for neuronal (TUBB3, DCX, and MAP2) and glial (GALC, GFAP, and S100B) markers of 146b-5p, hsa-miR-23b, hsa-miR-99a mimic transfected hNSCs and expressed as fold change compared with GFP transfected hNSCs (control). Statistical analysis was performed against transfected control; ± SDMs, *p < 0.05, **p < 0.001, ***p < 0.005, Student’s t-test.

Based on array analysis we selected three up-regulated miRNAs to substantiate their effects on neuronal/glial differentiation. Hsa-miR-99a, hsa-miR-146b-5p, and hsa-miR-23b mimics were transfected into hNSCs. Quantification of each miRNA mimic up-take and expression of GALC, GFAP, TUBB3, S100B, DCX, and MAP2 mRNAs were measured by qRT-PCR. All selected mimic miRNAs were appropriately transfected and quantified in hNSCs (Figure 4A) and they significantly increased GFAP, DCX, and MAP2 markers when compared to control. In contrast significant increases in gene expression of the oligodendrocyte precursor marker GALC and the astrocytic glial marker S100B were solely induced by hsa-miR-99a, whereas the neuronal precursor marker TUBB3 was solely induced by hsa-miR-146b-5p (Figure 4B).

### MiRNA target prediction analysis, validated by real-time RT-PCR, dual luciferase reporter assay, and miRNA KEGG pathway analysis using the DIANA Lab algorithms

**Figure 5:**
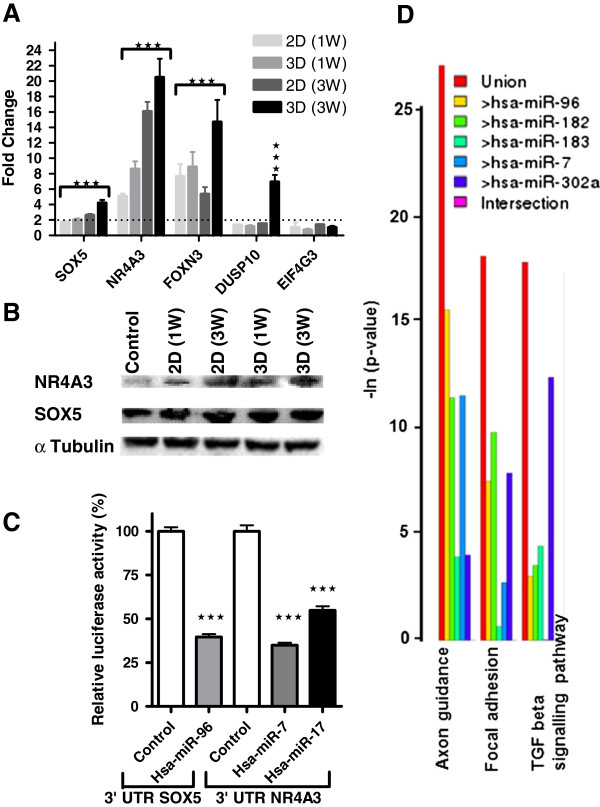
**Validation of miRNA target-predicted genes. (A)** qRT-PCR analysis performed on 1W and 3W differentiated hNSCs cultured on 2D and 3D substrates and expressed as fold change compared with proliferative control. Statistical analysis showed significant difference compared with control; ±SDMs, *P < 0.05, **P < 0.001, ***P < 0.005, Student *t* test. **(B)** SOX5 and NR4A3 protein quantification performed by Western blot on 1W and 3W differentiated hNSCs cultured on both 2D or 3D substrates and proliferative control. **(C)** Dual luciferase report assay. Measurement of the relative luciferase activity of SOX5 and NR4A3 3′-UTR constructs transfected with hsa-miR-96, and hsa-miR-7 and 17, respectively. Data are expressed as mean values ± SDMs and are shown as percentage of control (cells transfected with either SOX5 or NR4A3 3′-UTR constructs and control microRNA). Each bar represents values from three independent experiments, measured in triplicate. The relative activity of firefly luciferase expression was normalized to renilla luciferase activity. Data were analyzed with Student *t* test, ***P < 0.005. **(D)** MiRNA KEGG pathway analysis results obtained by using DIANA Lab.

Top ranking down-regulated miRNAs were selected to identify predicted miRNA target mRNAs by using the online tool, DIANA-microT 3.0. Although this analysis is constrained in terms of predictive ability, it is the only tool available for putative miRNA targets, and the output is useful for predicting hypothetical connections between miRNAs, targeted pathways, and biological functions. Since different algorithms often yield different results, the resulting putative target mRNAs were also checked with other similar online available algorithms: PicTar, and TargetScan (Table 3). Overall the number of resulting target sites was quite high, ranging from 1955/973 to 302/190 total target sites per gene identified. To overcome this issue we only investigated top ranked miRNA target genes that were previously referenced to brain development and function or were target sites of multiple investigated miRNAs. Validation of predicted targets was performed by qRT-PCR. SOX5, NR4A3 (NOR1), and FOXN3 were found to be significantly up-regulated in differentiated samples compared with control. In addition DUSP10 was only found to be up- regulated in 3D differentiated cultures at 3W. Among the putative target mRNAs, only EIF4G3 was not up-regulated and therefore did not correlate with the prediction (Figure 5A). To support miRNA target prediction we also examined protein expression of SOX5 and NR4A3.

**Table 3:** Computational target gene predictions

		**DIANALAB microT v3.0**					**PicTar**	**TargetScan**
**miRNA**	**Target gene**	**Rank**	**miTG score**	**Precision**	**SNR**	**Target sites/genes found**	**Score**	**Aggregate P**_**CT**_
**hsa-miR-96**	**SOX5**	2	64.73	1	7.3	1214/763	6.74	0.94
**hsa-miR-183**	**DUSP10**	1	26.44	0.72	1.62	302/190	4.75	0.85
**hsa-miR-302a**	**NR4A3**	27	19.86	0.84	6.22	863/473	3.05	NF
**hsa-miR-182**	**FOXN3**	3	37.4	0.94	6.87	1358/794	NF	0.96
**hsa-miR-7**	**NR4A3**	NF	NF	NF	NF	NF	1.61	0.17
**hsa-miR-7**	**FOXN3**	7	14.08	0.17	1.03	399/136	NF	0.88
**hsa-miR-20a**	**NR4A3**	9	26.13	0.85	9.44	1650/841	5.87	0.63
**hsa-miR-20b**	**NR4A3**	11	25.60	0.9	9	1955/973	5.87	0.63
**hsa-miR-17**	**NR4A3**	13	25.85	0.9	7.94	1928/961	5.38	0.63 + 0.37
**hsa-miR-20a**	**EIF4G3**	3	55.29	0.97	9.44	1650/841	NF	NF
**hsa-miR-20b**	**EIF4G3**	1	46.9	0.94	8	1955/973	NF	NF
**hsa-miR-17**	**EIF4G3**	1	46.87	0.94	7.94	1928/961	NF	NF

List of miRNA target-prediction mRNAs. MiRNAs, and target-predictive genes were identified by algorithms analysis by using Diana Lab, PicTar, and TargetScan. Scores (aggregate) are provided for each analysis.

We performed Western blot analysis for two of the predictive target mRNA, SOX5, and NR4A3 (NOR1). The differential expression of these proteins was verified in 2D and 3D differentiated hNSCs at both time points (1W and 3W) and compared with proliferative controls (Figure 5B).

Computational analysis of SOX5 3′-UTR with TargetScan revealed three putative binding sites for hsa-miR-96, located at nucleotides 347–375, 693–721, and 1,677–1,705, respectively. Same analysis of NR4A3 3′-UTR revealed three putative binding sites for hsa-miR-7 located at nucleotides 490–518, 568–596, and 2,612–2,640, and for hsa-miR-17, located at nucleotides 262–290, 1,556–1,584, and 1,688–1,716, respectively. To study the direct interaction between the miRNAs and their putative site on the 3′-UTR we used two commercially available plasmids containing either SOX5 or NR4A3 3′-UTR inserted downstream of the firefly luciferase reporter gene, and renilla luciferase gene for normalization. In HeLa cells transiently transfected with the SOX5 3′-UTR, and NR4A3 3′-UTR constructs and selected mimic miRNAs or miRNA control, a significant inhibition of luciferase activity was observed. Hsa-miR-96 caused a decrease in SOX5 3′-UTR luciferase activity by 60.34% ± 4.79%, and both hsa-miR-7 and hsa-miR-17 caused a decrease in NR4A3 3′-UTR luciferase activity by 65.01% ± 4.07% and 45.11% ± 6.76, respectively, compared with controls (Figure 5C).

Top-ranked downregulated miRNAs: hsa-miR-96, hsa-miR-182, hsa-miR-183, hsa-miR-7 and hsa-miR-302a were analyzed by using the DIANA-microT 4.0 algorithm to investigate the KEGG pathway. Axon guidance exhibited the highest union combined score (Figure 5D). This result mirrored axon-outgrowth measurements (Figure 2F).

## Discussion

Highly orchestrated programs of gene expression act to shape the developing nervous system. More recently, it has become clear that gene expression can also be modulated by several classes of small RNAs. In this study, we profiled miRNA differential expression patterns in hNSCs differentiated in 2D and 3D culture systems. HNSC differentiation was assessed by measuring processes/axons outgrowth and gene expression of well-established neural and glial markers. MiRNA profile of 2D versus 3D was very similar in terms of miRNA types; but the degree and timing of miRNA differential regulation was significantly different for miRNAs involved in both maintenance of stemness (hsa-miR-302a), and cell proliferation (the clusters miR-17-92 and miR-96-183). The upregulated miRNAs: hsa-miR-146b-5p, hsa-miR-23b, and hsa-miR-99a were selected, and their mimics were transfected into hNSCs to validate their correlation with differentiation of progenitors into neurons or glia. GALC and S100B gene expression was significantly upregulated after transfection of hsa-miR-99a mimics, suggesting a role in promoting differentiation of hNSC into glial restricted progenitors and oligodendrocyte progenitor cells. Similarly, hsa-miR-146b-5p transfection promoted upregulation of TUBB3 gene expression. Taken together, hsa-miR-99a and hsa-miR-146b-5p appear to be involved in early-stage commitment of glial and neuronal precursors, respectively.

The most significantly downregulated miRNAs were selected and analyzed to assess their putative target mRNAs with the intent of determining their functionality and identifying KEGG pathway maps for biological interpretation. By using online-available software analysis tools (DIANA Lab, PicTar, and TargetScans), we identified and verified a set of target mRNAs correlated with selected downregulated miRNAs. A single miRNA can recognize hundreds of targets. However, several miRNAs can target one gene. NR4A3, a target of the hsa-miR-7, hsa-miR302a, and miR-17 family, is a member of the NR4A family of nuclear receptors, which, depending on their level of expression, are involved in the differentiation, survival, apoptosis, and regulation of hippocampal axon guidance [50]. Similarly, SOX5, a target of hsa-miR-96, is reported to control cell-cycle progression in neural progenitors [51], axon length [52], migration, postmigratory differentiation, and projections of neurons [53]. NR4A3 and SOX5 were selected as possible candidate regulators of cell-cycle progression and axonal regulation.

QRT-PCR and Western blot analysis confirmed upregulation of both NR4A3 and SOX5. Furthermore, SOX5 and NR4A3 were identified as direct target genes of hsa-miR-96, and hsa-miR-7 and 17, respectively, by luciferase reporter assays. These findings, together with miRNA KEGG pathway analysis, support a possible regulatory role of these identified miRNAs as cellular regulators of axon guidance and outgrowth regulation. By mimicking a tissue-like environment, 3D scaffolds may enhance environmental guidance cues that modulate miRNA expression to promote axon outgrowth.

## Conclusions

Overall, 3D hNSC culture was associated with greater arrest of cell proliferation, increased incidence of differentiation, and differential degree and timing of regulation in miRNA expression compared with 2D systems. These observations suggest that 3D surface topography influences hNSC molecular behavior by modulating miRNAs associated with cell proliferation and stemness maintenance, thereby promoting cell differentiation.

Additionally, 3D architecture may provide contact guidance that could regulate miRNAs involved in developmental processes, including growing axons and neuritogenesis.

## Abbreviations

1W: 1 week; 2D: t-o dimensional; 3′-UTR: 3-prime untranslated region; 3D: three-dimensional; 3W: 3 weeks; 4-OHT: 4-hydroxytamoxifen; bFGF: basic fibroblast growth factor; DCX: doublecortin; EGF: epidermal growth factor receptor; GALC: galactosylceramidase; GFAP: glial fibrillary acidic protein; HNSC: human neural stem cell; Hsa-miR: human miRNA; ICC: immunocytochemistry; MAP2: microtubule-associated protein 2; miRNA: microRNA; QRT-PCR: real-time reverse-transcription PCR; S100B: S100 calcium-binding protein B; TUBB3: tubulin, beta 3 class III.

## Competing interests

All authors are employees, stock and/or stock-option holders in ReNeuron Ltd or its parent company.

## Authors’ contributions

LS carried out cell culture, immunocytochemistry, PCR analysis, acquisition and analysis of the miRNA data, immunocytochemistry, and Western blot, and conceived of the study and drafting of the manuscript. JDS assisted in the conception and design of the study and drafting of the manuscript. Both authors read and approved the final manuscript.
